# Cancer: Stress Link Redefined

**Published:** 2008-02

**Authors:** Bob Weinhold

As the battle against cancer continues, doctors, researchers, and patients continue to investigate new avenues for prevention and treatment. But the struggle isn’t getting any simpler. Researchers in a handful of laboratories have recently documented that, among many other triggers and promoters, stress hormones are a direct contributing factor in the progression of ovarian and nasopharyngeal cancer. Now a team from The Ohio State University has discovered a similar link with a third cancer, multiple myeloma.

Multiple myeloma, which involves lymphoid tumors, kills more than 10,000 Americans annually, usually within 3 to 4 years of diagnosis. Ovarian and nasopharyngeal cancers are characterized by solid epithelial tumors and result in about 15,000 and 650 U.S. deaths per year, respectively.

Until recently, stress hormones were thought to simply weaken the immune system and impair the body’s ability to fight cancer. Instead, the new evidence is showing that stress hormones such as norepinephrine, epinephrine, and cortisol, whether induced by psychological stimuli or environmental agents such as cold temperatures or infectious diseases, may play a much different role in cancer progression than generally presumed.

The Ohio State researchers began their study, published online 5 November 2007 ahead of print in *Brain, Behavior, and Immunity*, with a few givens: multiple myeloma has been linked with increased growth of supporting blood vessels, and vascular endothelial growth factor (VEGF) has been shown to play a role in that vessel growth. The team evaluated 3 human cell lines from 3 patients, representing different stages of multiple myeloma, to see if either of 2 doses of norepinephrine played a role in the expression of VEGF in the cell lines.

Based on their assessment of selected adrenergic receptors in the cell lines, they found that norepinephrine stimulated all 3 cell lines. The FLAM-76 cell line taken from bone marrow, which represented the earliest stage of multiple myeloma, showed the most significant stimulation.

Responses in all 3 cell lines were dose- and time-dependent, but there were no distinct patterns. Depending on the circumstances, either the higher or lower dose induced the most stimulation or suppression, and there were large differences over 1-, 3-, 6-, and 24-hour periods.

Eric Yang, lead author of the study and a research scientist at The Ohio State University Institute for Behavioral Medicine Research, points out that the effect on multiple myeloma of chronic stress from single or multiple sources is unknown. In addition, he says that the lowest concentration of norepinephrine tested was 10 times higher than levels typically detected in stressed humans. However, he says the team’s attempts to test at more realistic concentrations were stymied by erratic results that were difficult to interpret. But analogous research on ovarian cancer has shown similar effects at realistic doses.

Anil Sood, a professor of gynecologic oncology and cancer biology at the University of Texas M.D. Anderson Cancer Center, says that for stress and cancer the effects of other variables such as age, sex, race, ethnicity, and genetic makeup also remain a puzzle, and that there is little information on how best to manage or treat chronic stress in order to reduce its potential effects on tumor growth. Another concern is that the cell lines were grown in a tissue culture that can’t represent what actually occurs in the body, says Bruce Rabin, a professor of pathology, psychiatry, and psychology at the University of Pittsburgh School of Medicine. However, Rabin says the evidence from the experiments is a good first indication of possible effects of stress hormones on multiple myeloma.

